# Antibiotic residues in cattle and sheep meat and human exposure assessment in southern Xinjiang, China

**DOI:** 10.1002/fsn3.2568

**Published:** 2021-09-13

**Authors:** Yu Zhang, Jianjiang Lu, Yujun Yan, Jinhua Liu, Manli Wang

**Affiliations:** ^1^ School of Chemistry and Chemical Engineering/Key Laboratory of Environmental Monitoring and Pollutant Control of Xinjiang Bingtuan Shihezi University Shihezi China

**Keywords:** antibiotic residues, cattle and sheep meat, dietary exposure assessment, food safety, southern Xinjiang

## Abstract

In recent years, antibiotics have become widely used in animal breeding. The application of antibiotics in livestock may lead to the presence of antibiotic residues in animal‐derived foods, especially meat, that may pose a threat to human health. In this study, 26 common antibiotics (eight sulfonamides, nine fluoroquinolones, four tetracyclines, and five macrolides) were screened in 88 meat samples (cattle muscles and sheep muscles, kidneys, and livers) obtained from southern Xinjiang. The antibiotics were screened via the clean‐up step based on solid‐phase extraction and determined through ultraperformance liquid chromatography–tandem mass spectrometry. Moreover, their risk to human health was analyzed. Overall, 16 antibiotics were detected with a total detection rate of 95.46%. The percentage of noncompliant samples was 28.41% with an exceedance maximum residue limit of 1.14%. The illegal use rate of the antibiotic norfloxacin was 27.27%. The estimated daily exposure doses of all compounds in adults were <102.218 ng/kg bw/day even after applying the worst‐case scenario approach. This result demonstrated that the antibiotic residues in the tested samples imposed negligible harm to people's health and had an acceptable level of food safety risk. However, the high detection frequencies found in this work indicated that the risk of antibiotic residues could not be ignored given the cumulative risk of antibiotics, particularly the emergence of bacterial resistance, to the human body. The need for effective strategies and publicity for the judicious use of antibiotics to safeguard residents’ health is immediate.

## Graphical abstract

Antibiotic residues in meat may lead to several adverse health effects, such as the development of multidrug‐resistant microbial strains, allergic and anaphylactic reactions, and the disruption of normal intestinal flora. In southern Xinjiang, cattle and sheep meats are popular among the local people because of the influence of the eating habits of ethnic minority residents. Therefore, the monitoring of antibiotic residues in meats from southern Xinjiang warrants particular attention. The results of this study could contribute to the understanding of relevant departments and researchers of the concentration and residue status of antibiotics in commercial cattle and sheep meats from southern Xinjiang. In addition, it could provide data support for source analysis and risk assessment, as well as a scientific basis for the control of antibiotic pollution sources. The results of this study also suggested that people need to reduce their dependence on antibiotics and look for alternatives to decrease drug risks.

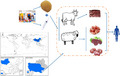

## 1 INTRODUCTION

Antibiotics have become the pillar of modern medicine since the discovery of penicillin in 1928 (Kumar et al., 2012). They are a class of secondary metabolites that are produced by microorganisms. They are chemically synthesized or semisynthesized analogous compounds that can inhibit the growth and survival of other microorganisms (Demain & Sanchez, 2009). In recent years, antibiotics have become widely used in animal husbandry to prevent and treat diseases and promote animal growth (Gelband et al., 2015). They are critical for meeting the rising meat demand of the growing population (Crawford, 1985). Global estimations suggest that ~45, 148, and 172 mg/kg of antibiotics are required per head of cattle, chicken, and pig produced each year, respectively. Moreover, the global livestock consumption of antibiotics in 2030 is forecasted to increase by 67% from that in 2010 (Van Boeckel et al., 2015). In China, a considerable demand for meat exists due to the country's large population. Concentrated animal feeding operations have emerged to fulfill this demand; these operations increase the density of food‐animal breeding, the spread of infectious diseases, and the use of antibiotics (Van Boeckel et al., 2015; Zhang et al., 2015). Previous studies have shown that the heavy and continuous use of antibiotics can lead to the deposition of antibiotic residues (Sanz et al., 2015) in chicken (Yang et al., 2020), beef (El‐Ghareeb et al., 2019), and pork (Kyriakides, Panderi, et al., 2020).

Antibiotics are one of the main veterinary drugs that have the potential to contaminate foods of animal origin. Furthermore, their residues in foods of animal origin, especially meats, may lead to several adverse health effects, such as the development of multidrug‐resistant microbial strains (Chang et al., 2015; Kjeldgaard et al., 2012), allergic and anaphylactic reactions (Baynes et al., 2016), and the disruption of normal intestinal flora (Cotter et al., 2012). In addition, bacterial resistance genes derived from animal microbiomes may be horizontally transferred to human microbiota and to human pathogenic bacteria (Marshall & Levy, 2011). Exposure assessment can provide sufficient knowledge to eliminate or minimize human exposure to veterinary antimicrobial agents (Bou Mitri et al., 2019). Therefore, the monitoring of veterinary drug residues in animals and animal‐derived foods warrant particular attention. Monitoring antibiotic residues in meats is crucial because these products are the main food consumed by humans.

Xinjiang is one of the five major pastoral areas in China. Southern Xinjiang, which is located to the south of the Tianshan Mountains, supports numerous ethnic minorities. Cattle and sheep meats are popular among local people because of the influence of the eating habits of ethnic minorities. However, few studies on the antibiotic residues and risk assessment of meats from southern Xinjiang have been conducted. Therefore, this study was designed to determine the concentrations of four types of antibiotics (eight sulfonamides, nine fluoroquinolones, four tetracyclines, and five macrolides) in the meat (cattle muscles and sheep muscles, kidneys, and livers) samples collected from southern Xinjiang. In this work, the concentrations of the most commonly utilized antibiotic agents for livestock purposes in the samples were determined by using ultraperformance liquid chromatography–tandem mass spectrometry (UPLC–MS/MS) method (Wang, Ren, et al., 2017). The analysis data were further combined with the consumption data on cattle and sheep meat in Xinjiang to calculate the ratio of the percentage of the estimated daily intake (EDI) to the acceptable daily intake (ADI). The objective of this study was to investigate whether the residues of antibiotics in cattle and sheep meats affect the health of residents in southern Xinjiang.

## 2 MATERIALS AND METHODS

### 2.1 Standards, chemicals, and materials

The antibiotic standards for sulfadiazine (SDZ, 99.9%), sulfamethoxazole (SMX, 99.9%), sulfamerazine (SMR, 99.5%), sulfamethazine (SM2, 99.9%), sulfamonomethoxine (SMM, 98.0%), trimethoprim (TMP, 99.9%), sulfaquinoxaline (SQX, 99.8%), sulfadimethoxine (SDM, 99.9%), tetracycline (TC, 99.0%), doxycycline (DXC, 99.9%), oxytetracycline (OTC, 98.0%), chlortetracycline (CLC, 81.7%), erythromycin (ERY, 95.4%), clarithromycin (CLA, 98.0%), azithromycin (AZI, 98.0%), roxithromycin (RTM, 97.0%), and tilmicosin (TIL, 94.0%) were purchased from Beijing Beina Chuanglian Biotechnology Institute (Beijing, China). Norfloxacin (NOR, ≥99%), enoxacin (ENO, ≥98%), ciprofloxacin (CIP, ≥98%), danofloxacin (DAN, ≥98%), enrofloxacin (ENR, ≥98%), fleroxacin (FLE, ≥98%), ofloxacin (OFL, ≥98%), sarafloxacin (SAR, ≥98%), and difloxacin (DIF, ≥98%) were purchased from Shanghai Yuanye Bio‐Technology Company (Shanghai, China). Methanol (MeOH, HPLC‐grade) and acetonitrile (ACN, HPLC‐grade) were purchased from Fisher Scientific (Pittsburgh, PA, USA). Formic acid (HPLC‐grade) was obtained from Anpel Company (Shanghai, China). Disodium ethylenediaminetetraacetate dihydrate (Na_2_EDTA, A.R.), disodium hydrogen phosphate (Na_2_HPO_4_·12H_2_O, A.R.), sodium dihydrogen phosphate dihydrate (NaH_2_PO_4_·2H_2_O, A.R.), sodium hydroxide (NaOH, A.R.), and citric acid monohydrate (C_6_H_8_O_7_·H_2_O) were purchased from Aladdin (Shanghai, China). Oasis HLB (6 cc, 200 mg) was provided by Waters (Milford, MA, USA). A nylon syringe filter (13 mm diameter, 0.22 μm) was purchased from Whatman (Maidstone, England). Ultrapure water (>18.2 MW/cm) was used in this study and was prepared in the laboratory.

EDTA–Mcllvaine buffer solution was prepared by dissolving 12.9 g of citric acid monohydrate, 10.9 g of disodium hydrogen phosphate, and 37.2 g of disodium ethylenediaminetetraacetate dihydrate in a 1000 ml volumetric flask with approximately 800 ml of water. The buffer was diluted to volume with water after adjusting the pH to 4.0 by using 1 mol/L sodium hydroxide.

### 2.2 Sampling

**FIGURE 1:**
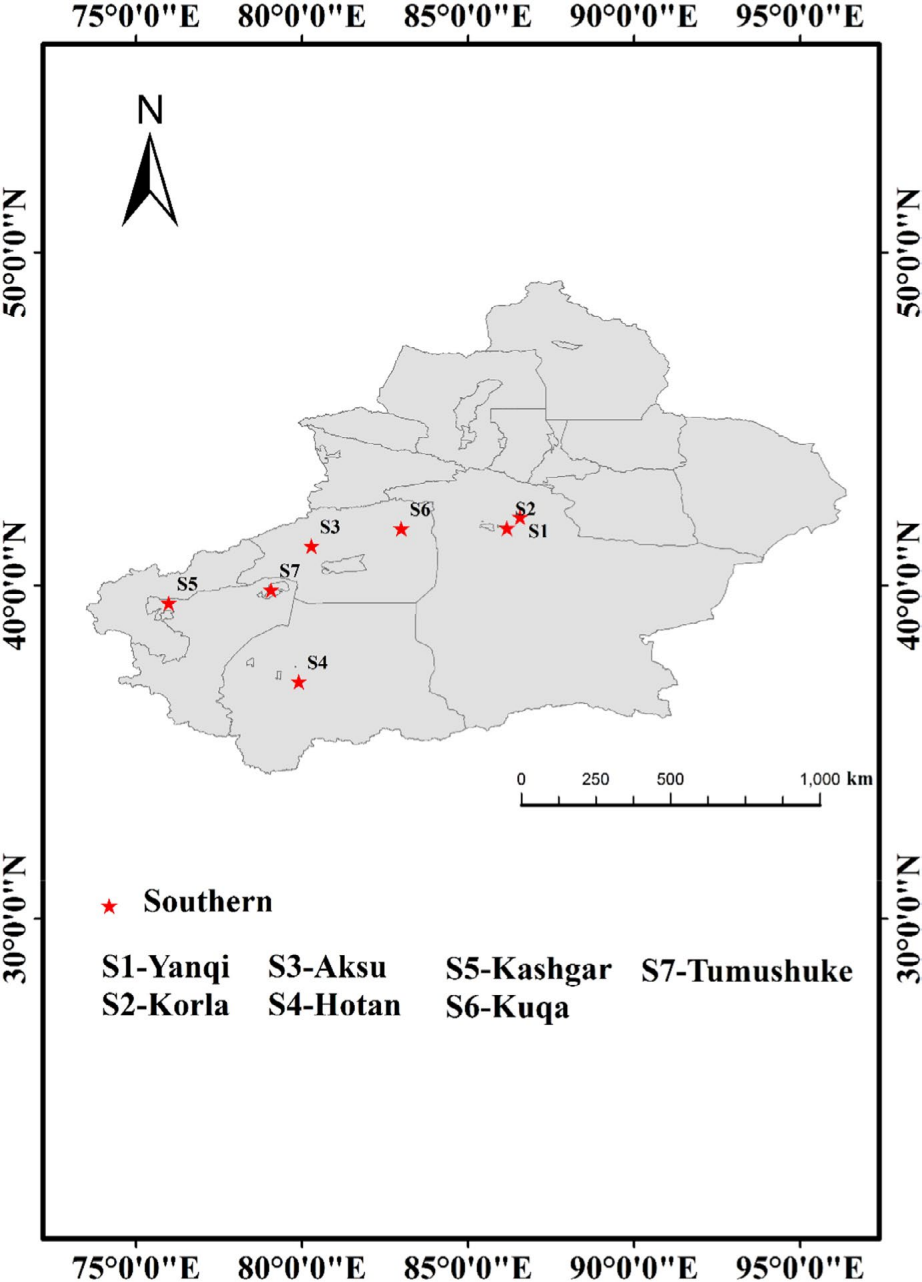
Distribution of the sampling sites in southern Xinjiang

A total of 88 samples, including 22 cattle muscles, 24 sheep muscles, 21 sheep livers, and 21 sheep kidneys, were collected from local supermarkets, retail stores, farmers’ markets, and farms in seven cities in Xinjiang from September 2020 to November 2020. The sampling points represented the major cities in the southern (S1–S7, *n* = 88) parts of Xinjiang (Figure 1). The four types of meat samples were selected as research objects because they are frequently consumed by Xinjiang residents.

Approximately 200–500 g of each sample was collected. Each sample was placed in a plastic container and immediately frozen after grinding and then stored at 18℃ until extraction. All the samples were analyzed within a week of collection.

### 2.3 Sample pretreatment and analysis

Sample pretreatment was performed via the solid‐phase extraction (SPE) method described by Zhang et al. and Pereira et al. (Pereira et al., 2018; Zhang et al., 2016). A 100 g portion of tissue was removed from each sample, minced, thoroughly mixed, properly labeled, and frozen at −20℃ until analysis. After defrosting, a portion of the sample (5.00 ± 0.01 g) was weighed in a 50 ml polyethylene centrifuge tube after it reached room temperature, after which 20 ml of 0.1 mol/L EDTA‐Mcllvaine buffer solution was added. Then, the sample was vortexed for 1 min, sonicated for 15 min, and then allowed to stand for 10 min away from light. Centrifugation was performed for 10 min at 2℃ and 13,000 × *g*, after which the supernatant was removed. Then, EDTA–Mcllvaine buffer solution (20 ml) was added to the pellets again, and the extraction procedure was repeated.

Extraction and clean‐up were performed through SPE with an Oasis HLB cartridge that was previously conditioned with 6 ml of MeOH followed by 6 ml of Milli‐Q water. The extract was applied at a flow rate of 2–3 ml/min. The filtrate was discarded, and the cartridge was washed with 2 ml of 5% MeOH aqueous solution. The column was drained and eluted with 6 ml of MeOH, and the eluent was collected. Finally, the eluent was evaporated to dryness at 45℃ under a gentle nitrogen stream, and the residue was redissolved in 1000 μl of MeOH.

LC–MS/MS was performed by using an UPLC–MS/MS system consisting of a Waters Acquity UPLC system, a XevoTQ‐S mass spectrometer (in positive‐ion multiple‐reaction monitoring mode), and a MassLynx4.1 workstation (Waters, Milford, MA, USA). The experimental conditions, including ESI–MS/MS and collision energy, are listed in Table S1.

Chromatographic separation was conducted with an ACQUITY UPLC BEHC18 column (50 mm × 2.1 mm, 1.7 μm, Waters, Dublin, Ireland). The injection volume was 1 μl, and the column temperature was maintained at 30℃. The mobile phases (0.1% (v/v) formic acid in water [A] and ACN [B]) were delivered at a flow rate of 0.3 ml/min. The initial solvent gradient was 14% B, which was increased over 2.5 min to 95% B and held for 1.5 min. Then, the gradient was decreased to 14% B and kept stable for 8 min to re‐equilibrate the column.

### 2.4 Quantification and quality control

Quantification was performed by using the matrix standard curve method. A 10‐point matrix‐matched standard (0.1, 0.5, 1, 5, 10, 20, 50, 100, 150, and 200 µg/L) was prepared by using serial dilutions of different blank matrices extract to verify linearity of different blank matrices. A blank was extracted then cleaned up by using SPE. Next, the standards were added, and several dilutions were performed to obtain 10 different concentrations. Linearity was confirmed on the basis of correlation coefficients (R^2^), which were higher than 0.990 for all analytes. The LOD was defined as the concentration of a target analyte that produced a peak with a signal‐to‐noise ratio (S/N) of 3. The limit of quantification (LOQ) was defined as S/N = 10. LODs were estimated to range between 0.0110 and 0.5212 μg/kg. Accuracy was expressed as recovery, which was determined by adding certain amounts of the antibiotic standard to the blank samples in triplicate. Precision was defined as the intraday relative standard deviation (RSD). The mean recovery rates for all target analytes in the sample spiked with three levels (5, 10, and 20 μg/kg) were in the range of 75.6%–117.6% with an RSD of <10%. Detailed information on the results of the equation, LOD, and recovery rate is provided in Table S2.

At the start of the experiment, all the laboratory equipment was alternately rinsed with ultrapure water and MeOH twice. Furthermore, new polypropylene centrifuge tubes were used. In addition, instrumental blanks and procedural blanks were injected with each batch of the sample, and the experimental results were deduced from the background noise.

### 2.5 Dietary exposure assessment

The daily exposure dose of antibiotics for adults was estimated on the basis of the mean daily consumption of sheep muscles, livers, and kidneys, and cattle muscles by using the following formula:

(1) $$\mathrm{EDI}=\frac{C_{c}\times M_{c}+C_{S}\times M_{S}+C_{L}\times M_{L}+C_{K}\times M_{K}}{M_{B}\times 1000}$$

where EDI is the estimated daily exposure dose (μg/kg bw/day); C_C_ is the antibiotic content in cattle muscle (μg/kg); *M_C_* is the daily adult consumption of cattle muscle (g/day); *C_S_* is the antibiotic content in sheep muscle (μg/kg); *M_S_* is the daily adult consumption of sheep muscle (g/day); *C_L_* is the antibiotic content in sheep liver (μg/kg); *M_L_* is the daily adult consumption of sheep liver (g/day); *C_K_* is the antibiotic content in sheep kidney (μg/kg); *M_K_* is the daily adult consumption of sheep kidney (g/day); and *M_B_* is the average body weight (kg) (Wang, Ren, et al., 2017). The average body weight (*M_B_*) of adults in Xinjiang is estimated to be 60 kg (Wang, Lu, et al., 2017). The details of the amount of cattle and sheep muscle consumption have also been reported in the 2019 Xinjiang Statistical Yearbook (Gao & Han, 2019). Given the lack of data on daily intake values from China, the consumption data for animal livers and kidneys were calculated from the ratio of liver or kidney weight to muscle weight (Zhang et al., 2010). The daily consumption rates for cattle muscle and sheep muscle, liver, and kidney calculated by using total muscle, liver, and kidney consumption were 13.86, 38.45, 0.96, and 0.19 g/day, respectively.

The EDI and ADI values of the antibiotic residues were used to calculate the %EDI/ADI ratio in accordance with the equation.

(2) $$\%\;\mathrm{EDI}\;\mathrm{to}\;\mathrm{ADI}\;\mathrm{ratio}=\frac{\mathrm{EDI}}{\mathrm{ADI}}\times 100.$$

Each ADI value is the maximum amount of a substance that a person can consume daily throughout his/her life without posing any risk. A %EDI/ADI ratio of <100 indicates that the EDI of antibiotics is lower than the acceptable daily intake, that is, no risk exists (Kyriakides, Lazaris, et al., 2020). When the concentration of an antibiotic was below the LOD, the EDI value was calculated under the assumption that the concentration of the antibiotic was zero. The EDI values and %EDI/ADI ratios were calculated by using the mean, and the highest concentrations found were applied to set the average approach and the worst‐case scenario approach (Pereira et al., 2018).

The maximum residue limits (MRLs) and regulations issued by the Ministry of Agriculture of the People's Republic of China (China, 2002) were used to assess the health risks associated with antibiotic residues. The ADI values were also obtained from the Ministry of Agriculture of the People's Republic of China (China, 2002). When available, the MRLs and ADIs are provided in Table 1. Some values are unavailable. The detection frequency was calculated as the number of samples detected/total number of samples × 100. The monitoring sample test results (concentration) were compared with the MRLs and used to determine whether the antibiotic residues in the sample exceeded the MRLs standard. The exceeding MRLs standard rate was calculated as the number of excess samples/total number of samples × 100.

**TABLE 1:** Maximum residue limits (MRLs) and acceptable daily intake (ADI) of meat

Antibiotics	MRL (μg/kg)			ADI (μg/kg bw/day)
	Muscle	Kidney	Liver	
Sulfonamides				
Sulfadiazine^f^	100	100	100	50
Sulfamethoxazole^f^	100	100	100	50
Sulfamerazine^f^	100	100	100	50
Sulfamethazine	100	100	100	50
Sulfamonomethoxine^f^	100	100	100	50
Trimethoprim^a^	50	50	50	4.2
Sulfaquinoxaline^f^	100	100	100	50
Sulfadimethoxine^f^	100	100	100	50
Fluoroquinolones				
Norfloxacin^b^	NA	NA	NA	NA
Enoxacin	NA	NA	NA	NA
Ciprofloxacin	NA	NA	NA	NA
Danofloxacin	200	400	400	20
Enrofloxacin^c^	100	200	300	6.2
Fleroxacin	NA	NA	NA	NA
Ofloxacin^b^	NA	NA	NA	NA
Sarafloxacin^d^	10	80	80	0.3
Difloxacin	400	800	1400	10
Tetracyclines				
Tetracycline^e^	200	1200	600	30
Doxycycline^e^	100	300	600	3
Oxytetracycline^e^	200	1200	600	30
Chlortetracycline^e^	200	1200	600	30
*Macrolides*				
Erythromycin	100	100	100	0.7
Clarithromycin^e^	NA	NA	NA	NA
Azithromycin^e^	NA	NA	NA	NA
Roxithromycin^e^	NA	NA	NA	NA
Tilmicosin	100	300	1000	40

Note

NA: not available.

^a^ Due to being usually used with sulfonamides in practice, trimethoprim was combined with sulfonamides.

^b^ According to the 2292 Bulletin issued by Ministry of Agriculture of the People's Republic of China (China, 2015), ofloxacin and norfloxacin cannot used in food animals.

^c^ The MRL of enrofloxacin is established for the sum of enrofloxacin and ciprofloxacin (China, 2002).

^d^ Exclusively used in chicken or turkey (China, 2002);

^e^ Exclusively used in human (Wang, Ren, et al., 2017).

^f^ Including the parent drug or its compounds (China, 2002).

^g^ The MRLs of Sulfonamides are for the sum of them (China, 2002).

## 3 RESULTS AND DISCUSSION

**FIGURE 2:**
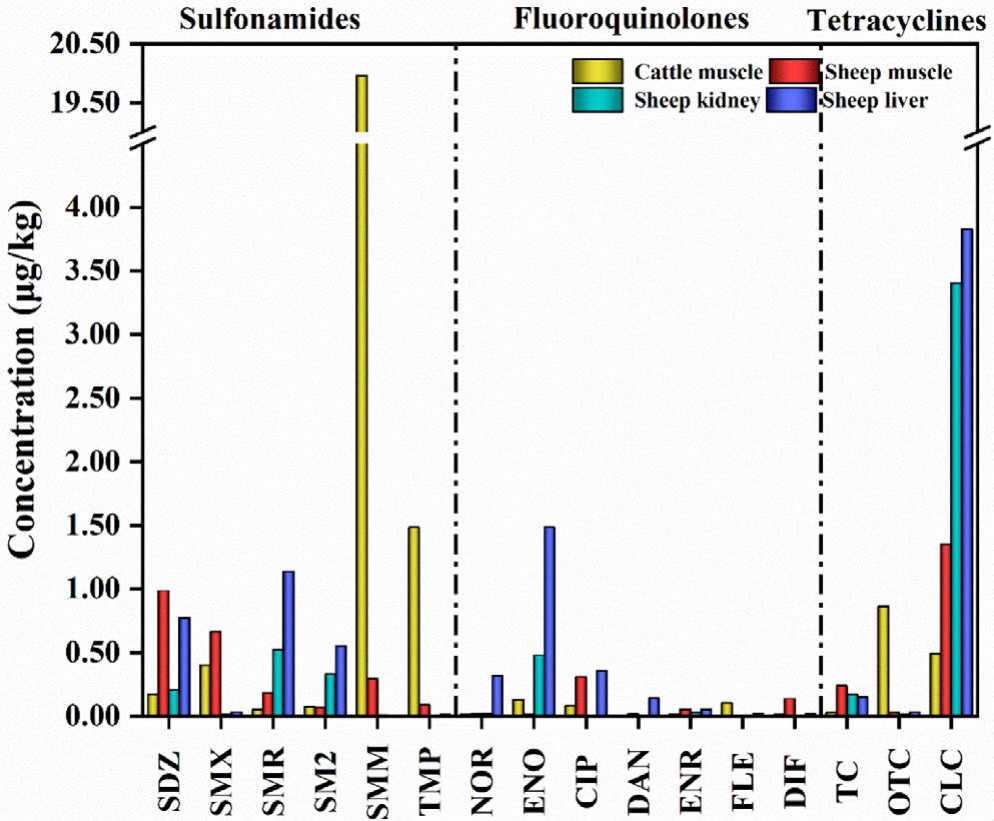
Mean concentrations of the 16 detected antibiotics (SDZ: sulfadiazine; SMX: sulfamethoxazole; SMR: sulfamerazine; SM2: sulfamethazine; SMM: sulfamonomethoxine; TMP: trimethoprim; NOR: norfloxacin; ENO: enoxacin; CIP: ciprofloxacin; DAN: danofloxacin; ENR: enrofloxacin; FLE: fleroxacin; DIF: difloxacin; TC: tetracycline; OTC: oxytetracycline; CLC: chlortetracycline. Due to being usually used with sulfonamides in practice, trimethoprim (TMP) was combined with sulfonamides.)

The concentrations of all 26 antibiotics were measured, and the aggregated data for each antibiotic are summarized in Table 2 and Figure 2. Of the 26 selected antibiotics, 16 were detected with a detection frequency of 5.68%–42.05% in 88 samples from seven cities in southern Xinjiang. The total detection frequency of these antibiotics residue was 95.46% in all samples. However, the concentrations of antibiotics in the samples were low. Most of the antibiotics, except for sulfamonomethoxine, which had a high mean concentration of 19.95 μg/kg, were present at concentrations below 4 μg/kg. The concentration of sulfamonomethoxine (424.40 μg/kg) detected in a cattle muscle sample exceeded the MRL with a detection frequency of 1.14% and 24 samples (27.27%) contained antibiotics that are not allowed in animals for human consumption.

**TABLE 2:** Frequency (%) and the maximum concentration (μg/kg) of the selected antibiotics (sulfaquinoxaline, sulfadimethoxine, ofloxacin, sarafloxacin, doxycycline, erythromycin, clarithromycin, azithromycin, roxithromycin, and tilmicosin were not detected)

Antibiotics	All samples (*n* = 88)		Cattle muscle (*n* = 22)		Sheep muscle (*n* = 24)		Sheep kidney (*n* = 21)		Sheep liver (*n* = 21)	
	Max (μg/kg)	DF (%)	Max (μg/kg)	DF (%)	Max (μg/kg)	DF (%)	Max (μg/kg)	DF (%)	Max (μg/kg)	DF (%)
**Sulfonamides**	424.40	80.68	424.40	54.55	19.01	75	2.22	95.24	11.52	100
Sulfadiazine	19.01	27.27	2.18	13.64	19.01	29.17	2.22	28.57	11.52	33.33
Sulfamethoxazole	7.76	34.09	2.18	31.82	7.76	41.67	0.18	14.29	0.48	14.29
Sulfamerazine	4.48	40.91	0.48	27.27	3.17	20.83	1.42	80.95	4.48	95.24
Sulfamethazine	1.49	42.05	1.36	27.27	1.06	25.00	0.98	85.71	1.49	90.48
Sulfamonomethoxine	424.40	9.09	424.40	13.64	6.52	8.33	0.18	4.76	0.04	4.76
Trimethoprim^g^	31.96	38.64	31.96	27.27	1.06	45.83	0.02	4.76	0.04	28.57
**Fluoroquinolones**	5.48	70.45	1.70	63.64	3.84	62.50	1.70	71.43	5.48	85.71
Norfloxacin	5.17	27.27	0.13	18.18	0.12	29.17	0.42	4.76	5.17	28.57
Enoxacin	5.48	26.14	1.70	9.09	0.18	8.33	1.70	61.90	5.48	80.95
Ciprofloxacin	4.12	25.00	0.74	22.73	3.84	29.17	ND	0.00	4.12	14.29
Danofloxacin	1.68	12.50	0.12	4.55	0.16	12.50	0.12	4.76	1.68	19.05
Enrofloxacin	1.06	11.36	0.16	9.09	1.06	12.50	0.2	23.81	0.68	9.52
Fleroxacin	1.14	5.68	1.14	9.09	ND	–	ND	–	0.16	14.29
Difloxacin	3.17	6.82	0.12	9.09	3.17	4.17	ND	–	0.24	9.52
**Tetracyclines**	23.76	68.18	16.64	81.82	23.76	75.00	14.96	61.90	21.32	52.38
Tetracycline	4.75	18.18	0.60	4.55	4.75	16.67	0.72	28.57	0.76	33.33
Oxytetracycline	16.64	43.18	16.64	77.27	0.2	37.50	0.16	9.52	0.36	14.29
Chlortetracycline	23.76	38.64	10.24	18.18	23.76	41.67	14.96	52.38	21.32	47.62

Abbreviations: DF, detection frequency; MAX, the maximum concentration; –, not calculated.

^a^ Due to being usually used with sulfonamides in practice, trimethoprim was combined with sulfonamides.

### 3.1 Types of antibiotic residues

Six out of eight sulfonamide antibiotics were detected, including sulfaquinoxaline and sulfadimethoxine. The residual concentration of sulfonamides ranged from not detected (ND) to 424.40 μg/kg. Among these antibiotics, sulfamethazine showed the highest detection frequency (42.05%), followed by sulfamerazine (40.91%). Among the nine fluoroquinolones, ofloxacin and sarafloxacin were not detected in the samples. The concentrations of the other detected antibiotics were between ND and 5.48 μg/kg. Norfloxacin had the highest detection frequency (27.27%), followed by enoxacin (26.14%). Three of the four tetracyclines, namely, tetracycline, oxytetracycline, and chlortetracycline, were detected. Their concentrations ranged between ND and 23.76 μg/kg. Oxytetracycline showed the highest detection frequency (43.18%), followed by chlortetracycline (38.64%) and tetracycline (18.18%). The total detection frequency of sulfonamides (80.68%) in all the samples was higher than that of fluoroquinolones (70.45%) and sulfonamides (68.18%). Macrolides were not detected in the samples. The exceedance rates of sulfonamides, fluoroquinolones, and tetracyclines were 1.14%, 0%, and 0%, respectively.

The use of antibiotics is a common way to prevent and treat diseases in livestock production and breeding. In the past, antibiotics were added to animal feed as growth‐promoting additives in animal production, especially in animal production under intensive cultivation conditions (Mund et al., 2017). However, in China, the use of antibiotic feed additives was banned on New Year's Day, 2020 (China, 2019). Therefore, only trace amounts of antibiotic residues are present in meat products (Ben et al., 2019). If the withdrawal time before the sale of meat is sufficient, antibiotics can be eliminated, and antibiotic residues can be present at negligible amount (Beyene, 2016). In this study, 16 antibiotics were detected in the samples from southern Xinjiang, and the rate of antibiotic use exceeding the standard was 1.14%. Norfloxacin, which has been prohibited for use in food animals (China, 2015), was found in the samples from southern Xinjiang at concentrations of up to 5.17 μg/kg and at the detection frequency of 27.27%. These results demonstrated that the overuse and illegal use of antibiotics exist. However, these results may also be caused by an insufficient withdrawal time before the sale or insufficient standard management after medication. In addition, the presence of more than four antibiotics in 34.09% of the samples was indicative of the probable overuse, cross‐use, and mixed use of antibiotics, especially the simultaneous use of multiple antibiotics, in the breeding process.

### 3.2 Antibiotic residues in different samples

In cattle muscle samples, the detection frequency of tetracyclines (81.82%) was the highest, followed by that of fluoroquinolones (63.64%) and sulfonamides (54.55%), which were both detected with concentrations between ND and 424.40 μg/kg. Oxytetracycline was the most frequently detected antibiotic in cattle muscle samples with the detection frequency of 77.27% and concentrations between ND and 16.64 μg/kg. The concentration of sulfamonomethoxine (424.40 μg/kg) detected in a cattle muscle sample exceeded the MRL. For sheep muscle samples, tetracycline (75.00%) and sulfonamides (75.00%) showed the highest detection frequencies, followed by fluoroquinolones (62.50%), which were present at concentrations of <23.76 μg/kg. Trimethoprim (45.83%) showed the highest detection frequency with a concentration range of ND to 1.16 μg/kg. Due to being usually used with sulfonamides in practice, trimethoprim was combined with sulfonamides. The detection frequency of sulfamethoxazole (41.67%) and chlortetracycline (41.67%) was lower than that of trimethoprim, and the concentrations of sulfamethoxazole and chlortetracycline ranged from ND to 7.76 μg/kg and ND to 23.76 μg/kg, respectively. In descending order, sulfonamides (95.24%), fluoroquinolones (71.43%), and tetracyclines (61.90%) were detected in sheep kidney samples at concentrations of <14.96 μg/kg. Sulfamethazine (85.71%) had the highest detection frequency, followed by sulfamerazine (80.95%). The concentrations of sulfamethazine and sulfamerazine ranged from ND to 0.98 μg/kg and ND to 1.42 μg/kg, respectively. Sulfonamides were detected in all sheep liver samples. The detection frequencies of fluoroquinolones and tetracyclines in sheep liver samples were 85.71% and 52.38%, respectively. The antibiotic residue concentrations in sheep liver samples were between ND and 21.32 μg/kg. Sulfamerazine (95.24%) showed the highest detection frequency, followed by sulfemethazine (90.48%). The concentrations of sulfamerazine and sulfemethazine ranged from ND to 4.48 μg/kg and from ND to 1.49 μg/kg, respectively. The antibiotics in sheep muscle, sheep kidney, and sheep liver samples did not exceed the MRLs.

Consistent with the results reported by El‐Ghareeb et al. (2019), the detection frequency of tetracycline was the highest in the cattle muscle samples. The detection frequencies of the antibiotics differed between liver–kidney and muscle samples because veterinary drug residues usually accumulate in the liver or kidneys rather than in the other tissues, and detection frequencies or residue levels differ depending on the site and route of administration (Doyle, 2006). Table 2 shows that the detection frequencies of various antibiotics in sheep livers were higher than those in sheep kidneys, which, in turn, were higher than those in sheep muscles. Yang et al. reported that the detection values and frequencies of antibiotic residues in chicken giblets (e.g., chicken liver and gizzards) were high (Yang et al., 2020). These results may be related to drug metabolism in organisms. Hepatocytes play a crucial role in the metabolism of foreign bodies, drugs, and xenobiotic compounds, some of which are toxic and ultimately processed by the kidneys (Beyene, 2016). Other factors, including the outbreaks of livestock diseases, an excessive demand for meat and meat products, the weak financial status and low education and expertise of livestock farmers, and the type of husbandry system (intensive and extensive), affect antibiotic use and residues (Alhaji et al., 2018). Although the detection frequencies of antibiotics in all the samples from southern Xinjiang were slightly high and the types of antibiotics used were mixed, the overall concentration level was low. These findings may be related to the production mode of cattle and sheep in Xinjiang (grassland grazing and farmhouse breeding) (Zhang et al., 2019). Despite the gradual transition to large‐scale production, the traditional household production method still occupies an important position in livestock farming in the region. This method involves different feeding and management conditions (Gan et al., 2014), which may complicate the use of antibiotics.

### 3.3 Dietary exposure assessment and uncertainties

**FIGURE 3:**
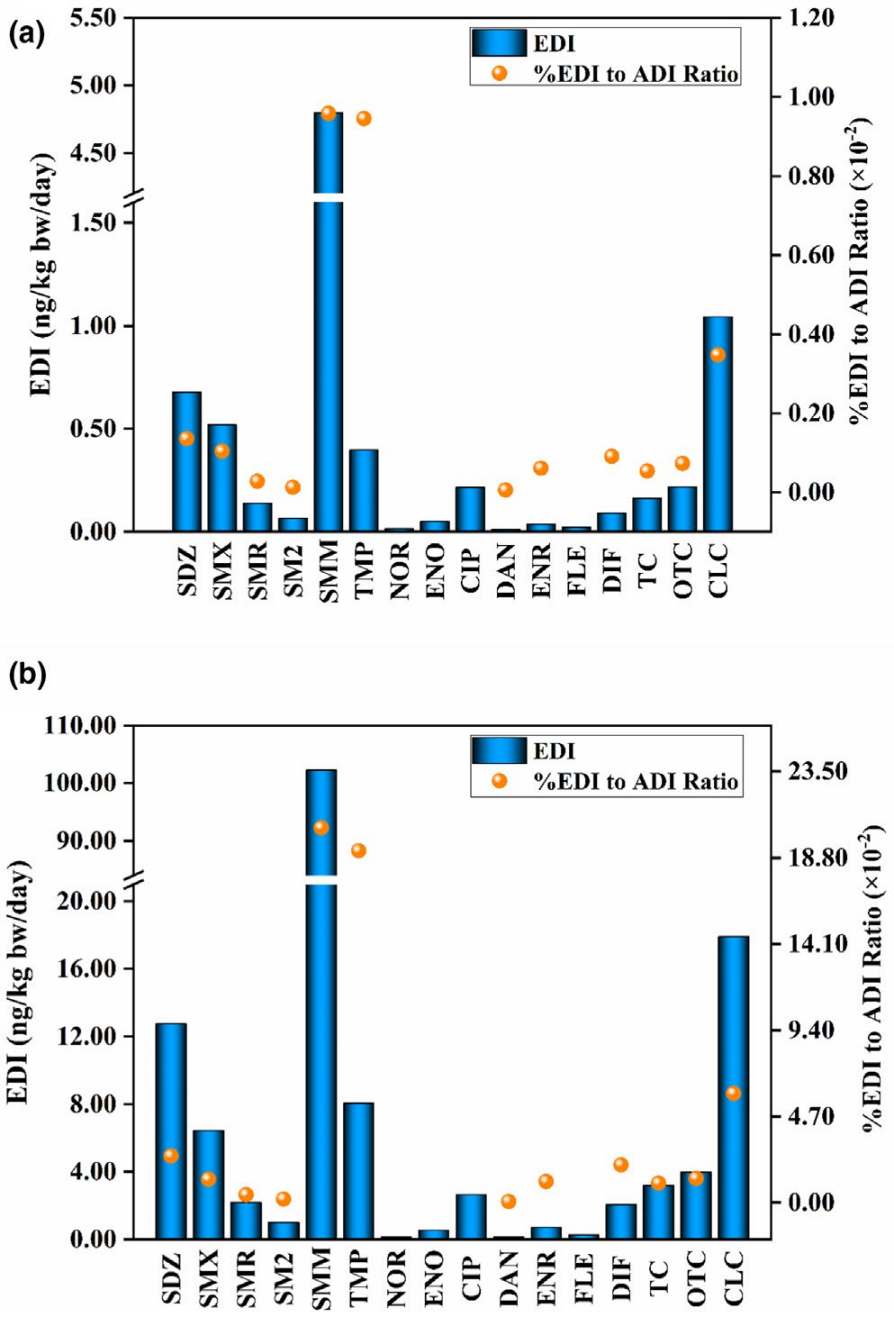
(a) Comparison of estimated daily intake (EDI) and % EDI to ADI ratio (using averages approaches); (b) comparison of EDI and % EDI to ADI ratio (considering worst‐case scenario) with respect to the samples of southern Xinjiang (SDZ, sulfadiazine; SMX, sulfamethoxazole; SMR, sulfamerazine; SM2, sulfamethazine; SMM, sulfamonomethoxine; TMP, trimethoprim; NOR, norfloxacin; ENO, enoxacin; CIP, ciprofloxacin; DAN, danofloxacin; ENR, enrofloxacin; FLE, fleroxacin; DIF, difloxacin; TC, tetracycline; OTC, oxytetracycline; CLC, chlortetracycline. For some ADI values that were unavailable, some values of % EDI to ADI ratio are not calculated; therefore, no data of them are displayed in Figure 3)

In this study, only cattle muscles and sheep muscles, livers, and kidneys were assumed to be consumed. For some ADI values that were unavailable, the data of antibiotics with the EDI value and %EDI/ADI ratio are shown in Figure 3, and all EDI values and %EDI/ADI ratio values are included in Table S4. Figure 3 and Table S4 show that the EDI range of sulfonamides in the southern Xinjiang samples was 0.065–4.799 ng/kg bw/day, and the corresponding %EDI/ADI ratio values ranged from 0.00013 to 0.00959. Sulfamonomethoxine was the most influential antibiotic with an EDI value of 4.799 ng/kg bw/day and a %EDI/ADI ratio value of 0.00959. Moreover, in the worst‐case scenario, its EDI value was 102.218 ng/kg bw/day, and its %EDI/ADI ratio value was 0.20444. For tetracyclines, the EDI range was 0.162–1.045 ng/kg bw/day, and the corresponding %EDI/ADI ratio range was 0.00054–0.00348. The most influential antibiotic was chlortetracycline, which had an EDI value and a %EDI/ADI ratio of 1.045 ng/kg bw/day and 0.00348, respectively. In the worst‐case scenario, its EDI value and %EDI/ADI ratio were 17.899 ng/kg bw/day and 0.05966, respectively. For fluoroquinolones, the EDI range was 0.013–0.216 ng/kg bw/day. The ADI values for norfloxacin, enoxacin, ciprofloxacin, and fleroxacin were unavailable. Danofloxacin, enrofloxacin, and difloxacin had %EDI/ADI ratios of 0.00006, 0.00061, and 0.00091, respectively, and %EDI/ADI ratios of 0.00069, 0.01160, and 0.02059, respectively, in the worst‐case scenario.

The EDI of sulfonamides (6.595 ng/kg bw/day) in the samples from southern Xinjiang was the highest, followed by that of tetracyclines (1.425 ng/kg bw/day) and fluoroquinolones (0.445 ng/kg bw/day). The %EDI/ADI values were far <100 for all antibiotics. These results showed that the EDI value that was calculated on the basis of dietary consumption was considerably lower than the ADI value even after considering the worst‐case scenario, indicating that human exposure to antibiotic residues through cattle muscle and sheep muscle, liver, and kidney consumption was low in southern Xinjiang.

The exposure assessment indicated that the health risk to the population was negligible. Residue control in southern Xinjiang should be based on risk analysis and consumer protection. This recommendation provides a reference for the establishment of a national unified food residue control standard.

Given that the dietary antibiotic intakes determined in this study only considered antibiotic exposure from cattle and sheep meat, they did not include total antibiotic dietary exposure. Other foods, such as other meats, milk, fish, vegetables, grains, and drinking water, as well as exposure to occupational or residential sources, may increase overall exposure rate.

### 3.4 Comparison with other studies

The results of this study were compared with the data from other studies that focused on the detection of antibiotic residues in meat. The detection frequency of sulfonamides in the samples of this study was higher than the 5.2% found for chicken and chicken giblet samples from Fujian (Yang et al., 2020). By contrast, no sulfonamides were detected in pork and chicken samples from Shanghai (Wang, Ren, et al., 2017). The highest detected concentration of sulfonamides in this study was 424.40 μg/kg, which was higher than the highest concentrations of 62 and 193 μg/kg found in chicken and chicken liver samples, respectively, from Malaysia (Cheong et al., 2010). The detection frequency of fluoroquinolones in the samples in this study was higher than that in the beef and chicken samples from Turkey (51.1%) (Er et al., 2013) and lower than that in chicken samples obtained from a Portuguese supermarket (77.8%) (Pereira et al., 2018). The maximum detected concentration of fluoroquinolones in the samples in this study (5.48 μg/kg) was drastically lower than that in Portuguese samples (147 μg/kg) (Pereira et al., 2018). The detection frequency of tetracycline in the samples in this study was higher than that in pork samples from Vietnam (5.52%) (Van Nhiem et al., 2006). Moreover, the highest concentration (23.76 μg/kg) of tetracycline found in the samples in this study was lower than that found in cattle samples from Saudi Arabia (92.82 μg/kg) (El‐Ghareeb et al., 2019) and pork samples from Cyprus (373 μg/kg) (Kyriakides, Panderi, et al., 2020).

To date, few studies have estimated the daily intake of antibiotic residues through food intake. Ji et al. focused on the consumption of drinking water, beef, pork, chicken, dairy products, and fish and revealed that the average daily intake of sulfamethazine, trimethoprim, enrofloxacin, and roxithromycin by the general population of Korea were 679.3, 88.8, 619.2, and 49.7 pg/kg bw/day, respectively (Ji et al., 2010). The estimated daily exposure dose of 20 antibiotics in samples from Shanghair, including livestock and poultry meats and aquatic products, was <1 μg kg^−1^ day^−1^ (Wang, Ren, et al., 2017). Doxycycline residues originating from the consumption of Cyprus pork meat showed the highest EDI values of 1.47 and 1.84 μg/kg bw/day for men and women, respectively (Kyriakides, Panderi, et al., 2020). The predicted single antibiotic EDI values based on the dietary consumption of children ranged from 2.2 × 10^−4^ μg/kg bw/day (ofloxacin) to 0.30 μg kg^−1^ day^−1^ (oxytetracycline) (Li et al., 2017). The EDI values in this study were lower than those in other studies.

## 4 CONCLUSION

SPE combined with UPLC–MS/MS was used to detect the residues of four types of antibiotics in meat samples obtained from southern Xinjiang. Antibiotic residues in the cattle muscle and sheep muscle, kidney, and liver samples collected from southern Xinjiang were subjected to risk assessment to provide data support for the supervision of the antibiotic residues present in meats from Xinjiang. A total of 88 samples were collected from southern Xinjiang, and 16 types of antibiotics were detected with a detection frequency of 95.46%. The percentage of noncompliant samples was 28.41% with an exceedance MRL rate of 1.14%. The illegal norfloxacin use rate was 27.27%. The levels of antibiotic residues in the kidney and liver samples were slightly higher than those in other samples, suggesting that the intake of animal viscera should be reduced. Compared with those in other studies, the samples from southern Xinjiang in this study were characterized by high detection frequencies, low concentrations, and the cross‐use of multiple antibiotics. Thus, the consumption of the samples from southern Xinjiang was associated with an acceptable level of food safety risk and does little harm to human health. However, the high detection frequencies found in this study indicated that the risk of antibiotic residues cannot be ignored because antibiotics have a cumulative effect, such as the emergence of bacterial resistance, on the human body.

## CONFLICT OF INTEREST

The authors declare no conflict of interest.

## AUTHOR CONTRIBUTIONS

Yu Zhang involved in conceptualization, methodology, data curation, formal analysis, investigation, and writing the original draft. Jianjiang Lu involved in conceptualization, funding acquisition, methodology, supervision. Yujun Yan involved in conceptualization and resources. Jinhua Liu and involved in investigation.

## Supporting information

Tables S1‐S4

## Data Availability

The data that support the findings of this study are available on request from the corresponding author. The data are not publicly available due to privacy or ethical restrictions, and the data that support the findings of this study are available in the supplementary material of this article.
