# Supplementary material for: Antibiotic residues in cattle and sheep meat and human exposure assessment in southern Xinjiang, China
Source: Food Sci Nutr. 2021 Sep 13;9(11):6152–61. doi: 10.1002/fsn3.2568 (PMC8565197; doi:10.1002/fsn3.2568)
Supplement: Supplementary file 1 — Tables S1‐S4 [file FSN3-9-6152-s001.docx]

**Supplementary Information**

**Antibiotic residues in cattle and sheep meat and human exposure assessment in southern Xinjiang, China**

Yu Zhang^a^, Jianjiang Lu^a^*, Yujun Yan ^a^*, Jinhua Liu^a^ and Manli Wang^a^

*^a^ School of Chemistry and Chemical Engineering, Key Laboratory of Environmental Monitoring and Pollutant Control of Xinjiang Bingtuan, Shihezi University, Shihezi (832003), Xinjiang, China*

Yu Zhang: E-mail address: ZhangYuAYYQX@163.com; Tel: +86 18290778807

*Jianjiang Lu: E-mail address: lujianjiang2015@163.com; Tel: +86 993 2055016; Fax: +86 993 2057270

*Yujun Yan: E-mail address: yyj_tea@shzu.edu.cn; Tel: +86 17590931776

Jinhua Liu: 18290779514@163.com; Tel: +86 18290779514

Manli Wang: wangmanli8090@163.com

**Table of Contents**

**Table S1.** MS conditions for the determination of 26 antibiotics.

**Table S2.** Equations, linearities, LODs, recovery rates, and RSD data for the investigated antibiotics.

**Table S3.** Mean concentration (μg/kg) of the selected antibiotics.

**Table S4.** Estimated daily intake values (EDI) and percentage of EDI to acceptable daily intake (ADI) ratio using averages and worst case scenario (wcs) approaches.

Table S1. MS conditions for the determination of 26 antibiotics

| Antibiotics | Retention time (min) | Precursor ion (m/z) | Product ion (m/z) | Cone voltage (V) | Collision  energy (eV) |
| --- | --- | --- | --- | --- | --- |
| Sulfadiazine | 1.76 | 250.92 | 155.89*/91.98 | 28 | 14/24 |
| Sulfamethoxazole | 3.06 | 253.91 | 155.89*/91.98 | 2 | 16/26 |
| Sulfamerazine | 2.44 | 264.94 | 91.99*/155.91 | 36 | 26/16 |
| Sulfamethazine | 2.7 | 278.94 | 185.93*/91.99 | 44 | 16/28 |
| Sulfamonomethoxine | 2.9 | 280.94 | 155.89*/91.98 | 36 | 16/28 |
| Trimethoprim | 2.64 | 291.03 | 229.94*/122.99 | 46 | 22/24 |
| Sulfaquinoxaline | 3.36 | 300.92 | 155.89*/91.98 | 38 | 16/28 |
| Sulfadimethoxine | 3.35 | 310.94 | 155.90*/91.99 | 50 | 20/30 |
| Norfloxacin | 2.65 | 319.9 | 276.0*/232.94 | 46 | 20/26 |
| Enoxacin | 2.61 | 320.89 | 233.92*/256.93 | 48 | 20/18 |
| Ciprofloxacin | 2.7 | 331.88 | 288.0*/314 | 40 | 22/22 |
| Danofloxacin | 2. 75 | 358.05 | 96.01*/314 | 54 | 2020 |
| Enrofloxacin | 2.81 | 359.92 | 316.0*/244.94 | 46 | 26/26 |
| Fleroxacin | 2.67 | 369.88 | 268.88*/193.92 | 24 | 26/44 |
| Ofloxacin | 2.67 | 362.01 | 260.88*/318 | 52 | 26/22 |
| Sarafloxacin | 2.95 | 385.88 | 284.88*/298.92 | 34 | 32/26 |
| Difloxacin | 2.98 | 399.95 | 298.87*/356 | 50 | 28/24 |
| Tetracycline | 2.81 | 444.99 | 409.97*/427 | 36 | 18/25 |
| Doxycycline | 3.25 | 445.04 | 428.2*/153.94 | 4 | 22/28 |
| Oxytetracycline | 2.7 | 460.98 | 425.96*/443.2 | 2 | 18/15 |
| Chlortetracycline | 3.16 | 478.93 | 443.91*/153.93 | 4 | 22/26 |
| Erythromycin | 3.65 | 734.32 | 158.02*/576.25 | 12 | 30/20 |
| Clarithromycin | 4.12 | 748.34 | 158.03*/590.26 | 18 | 28/18 |
| Azithromycin | 4.12 | 749.37 | 158.03*/591.29 | 16 | 38/30 |
| Roxithromycin | 4.16 | 837.33 | 158.02*/679.31 | 6 | 34/22 |
| Tilmicosin | 3.47 | 869.43 | 174.01*/88.01 | 76 | 42/64 |

*: quantitative ion.

Table S2. Equations, linearities, LODs, LOQs, recovery rates, and RSD data for the investigated antibiotics.

| Antibiotics | Matrix | Equation | Linearity (R^2^) | LOD (μg/kg) | LOQ (μg/kg) | Mean recoveries (%) | RSD (%) |
| --- | --- | --- | --- | --- | --- | --- | --- |
|  |  |  |  |  |  | 5/10/20 (μg/kg) | 5/10/20 (μg/kg) |
| SDZ | Cattle muscle | Y=4348.02x+1651.19 | 0.9998 | 0.1858 | 0.2601 | 97.45/90.43/97.96 | 4.86/2.45/3.88 |
|  | Sheep muscle | Y=4180.79x+1587.68 | 0.9994 | 0.1799 | 0.2519 | 96.32/93.22/92.34 | 3.76/2.21/1.89 |
|  | Sheep kidney | Y=3679.09x+1397.16 | 0.9990 | 0.2212 | 0.3097 | 95.19/96.01/98.96 | 2.66/1.97/1.99 |
|  | Sheep liver | Y=3846.33x+1460.67 | 0.9986 | 0.2314 | 0.3240 | 94.06/98.82/92.35 | 1.56/1.73/2.01 |
| SMX | Cattle muscle | Y=2698.24x-121.748 | 0.9992 | 0.0110 | 0.0154 | 87.53/85.44/84.39 | 2.42/1.23/1.19 |
|  | Sheep muscle | Y=2759.56x+1245.15 | 0.9996 | 0.0140 | 0.0196 | 95.46/84.85/91.22 | 5.56/3.46/4.55 |
|  | Sheep kidney | Y=2452.95x+110.68 | 0.9991 | 0.0170 | 0.0238 | 95.40/88.33/91.87 | 2.34/2.45/2.26 |
|  | Sheep liver | Y=2575.59x+116.214 | 0.9986 | 0.0190 | 0.0266 | 98.36/92.21/87.09 | 3.24/4.37/2.46 |
| SMR | Cattle muscle | Y=3708.28x-54.0253 | 0.9952 | 0.0937 | 0.1312 | 97.32/98.83/95.75 | 1.14/1.30/0.50 |
|  | Sheep muscle | Y=3973.16x+578.84 | 0.9979 | 0.0945 | 0.1323 | 89.35/87.80/90.90 | 4.22/5.33/4.98 |
|  | Sheep kidney | Y=3310.96x+48.237 | 0.9946 | 0.1001 | 0.1401 | 86.35/84.47/82.60 | 7.33/2.44/4.33 |
|  | Sheep liver | Y=3575.84x+52.0958 | 0.9953 | 0.0989 | 0.1385 | 90.09/78.80/84.39 | 4.38/3.55/3.45 |
| SM2 | Cattle muscle | Y=5536.43x+415.628 | 0.9985 | 0.0368 | 0.0515 | 93.34/91.36/95.54 | 4.67/1.30/1.91 |
|  | Sheep muscle | Y=5662.26x-425.07 | 0.9991 | 0.0411 | 0.0575 | 107.76/94.83/99.14 | 5.59/4.98/3.44 |
|  | Sheep kidney | Y=5033.12x+377.844 | 0.9979 | 0.0489 | 0.0685 | 102.95/91.51/96.08 | 6.34/4.57/4.44 |
|  | Sheep liver | Y=5410.60x+406.18 | 0.9981 | 0.0521 | 0.0729 | 94.56/91.06/87.56 | 7.65/6.54/4.58 |
| SMM | Cattle muscle | Y=4296.99x+159.66 | 0.9973 | 0.0333 | 0.0466 | 84.69/87.75/91.06 | 4.14/1.26/0.75 |
|  | Sheep muscle | Y=4423.37x-164.356 | 0.9982 | 0.0244 | 0.0342 | 91.13/87.75/84.38 | 5.89/2.44/3.99 |
|  | Sheep kidney | Y=3791.46x+1408.76 | 0.9967 | 0.0368 | 0.0515 | 93.03/85.28/77.53 | 8.54/7.76/4.71 |
|  | Sheep liver | Y=3854.65x+143.224 | 0.9972 | 0.0376 | 0.0526 | 86.39/82.07/86.39 | 6.94/6.99/4.99 |
| TMP | Cattle muscle | Y=4458.46x-161.417 | 0.9991 | 0.0137 | 0.0192 | 92.64/92.22/95.51 | 0.51/2.59/1.48 |
|  | Sheep muscle | Y=4374.34x+1583.71 | 0.9993 | 0.0112 | 0.0157 | 82.60/99.30/97.14 | 2.66/2.19/1.56 |
|  | Sheep kidney | Y=3785.48x-137.052 | 0.9990 | 0.0167 | 0.0234 | 84.39/103.60/90.62 | 8.43/4.33/3.43 |
|  | Sheep liver | Y=3869.61x+140.098 | 0.9978 | 0.0178 | 0.0249 | 99.06/93.98/91.60 | 5.66/4.88/3.09 |
| SQX | Cattle muscle | Y=6789.57x+1160.83 | 0.9983 | 0.1031 | 0.2196 | 88.23/92.98/93.16 | 0.12/2.34/2.64 |
|  | Sheep muscle | Y=7242.21x+1238.219 | 0.9985 | 0.1113 | 0.2371 | 81.71/97.57/90.35 | 2.34/2.11/1.30 |
|  | Sheep kidney | Y=5431.66x+928.664 | 0.9977 | 0.2143 | 0.4565 | 83.59/79.30/85.73 | 3.33/3.42/3.11 |
|  | Sheep liver | Y=5884.29x+1006.053 | 0.9956 | 0.2167 | 0.4616 | 102.10/85.08/88.49 | 3.21/3.45/1.22 |
| SDM | Cattle muscle | Y=14591.7x+1460.4 | 0.9987 | 0.2247 | 0.4786 | 89.25/88.37/88.84 | 2.05/3.57/0.32 |
|  | Sheep muscle | Y=13028.3x+1303.929 | 0.9967 | 0.2389 | 0.5089 | 91.72/85.99/81.21 | 9.37/2.12/2.34 |
|  | Sheep kidney | Y=10422.64x+1043.1 | 0.9957 | 0.3658 | 0.7792 | 96.64/84.03/86.13 | 4.77/3.91/2.34 |
|  | Sheep liver | Y=10943.78x+1095.3 | 0.9965 | 0.3579 | 0.7623 | 100.79/88.69/92.72 | 5.23/4.23/2.98 |
| NOR | Cattle muscle | Y=1430.42x-109.805 | 0.9978 | 0.0183 | 0.0295 | 86.03/91.77/87.99 | 8.54/1.58/0.57 |
|  | Sheep muscle | Y=1379.33x+1050.1 | 0.9984 | 0.0179 | 0.0288 | 80.93/93.58/86.22 | 2.29/1.72/1.22 |
|  | Sheep kidney | Y=1277.16x-98.04 | 0.9989 | 0.0221 | 0.0356 | 81.64/77.56/81.64 | 8.31/3.74/3.09 |
|  | Sheep liver | Y=1328.25x+101.962 | 0.9990 | 0.0278 | 0.0448 | 84.67/81.41/79.90 | 4.58/4.02/2.33 |
| ENO | Cattle muscle | Y=1639.81x-33.83 | 0.9954 | 0.1134 | 0.1826 | 85.53/85.66/85.72 | 4.01/4.98/0.17 |
|  | Sheep muscle | Y=1639.81x-201.11 | 0.9985 | 0.1067 | 0.1718 | 80.93/83.58/86.22 | 1.33/1.49/1.07 |
|  | Sheep kidney | Y=1537.32x+1172 | 0.9987 | 0.1247 | 0.2008 | 81.64/87.56/81.64 | 7.82/2.11/1.60 |
|  | Sheep liver | Y=1451.92x+29.954 | 0.9990 | 0.1239 | 0.1995 | 84.67/81.41/84.90 | 2.97/1.73/0.49 |
| CIP | Cattle muscle | Y=1354.17x+2588.5 | 0.9901 | 0.1228 | 0.1977 | 84.32/84.33/88.35 | 8.02/1.56/0.81 |
|  | Sheep muscle | Y=1384.26x+2646.022 | 0.9945 | 0.1312 | 0.2112 | 81.64/77.56/81.64 | 2.19/1.39/1.13 |
|  | Sheep kidney | Y=1203.71x+2300.9 | 0.9946 | 0.1379 | 0.2220 | 87.41/83.91/89.93 | 4.85/1.23/1.46 |
|  | Sheep liver | Y=1233.80x+2358.41 | 0.9965 | 0.1399 | 0.2252 | 90.51/90.34/87.32 | 3.50/1.06/1.78 |
| DAN | Cattle muscle | Y=1019.08x-212.375 | 0.9969 | 0.1111 | 0.1789 | 89.35/89.9/88.23 | 7.59/3.56/1.68 |
|  | Sheep muscle | Y=1234.17x+333.13 | 0.9979 | 0.1056 | 0.1700 | 90.79/75.66/78.68 | 5.94/3.27/1.29 |
|  | Sheep kidney | Y=1001.36x+208.682 | 0.9968 | 0.1165 | 0.1876 | 80.12/89.83/85.23 | 6.90/2.98/0.90 |
|  | Sheep liver | Y=1036.80x+216.068 | 0.9966 | 0.1187 | 0.1911 | 90.79/84.06/87.42 | 7.86/2.69/0.51 |
| ENR | Cattle muscle | Y=3003.571x-583.747 | 0.9964 | 0.0406 | 0.0654 | 86.35/90.41/93.8 | 1.92/0.58/1.33 |
|  | Sheep muscle | Y=3473.52x+667.22 | 0.9956 | 0.0412 | 0.0663 | 90.27/86.77/88.70 | 3.22/3.11/1.09 |
|  | Sheep kidney | Y=2938.28x+571.1 | 0.9967 | 0.0477 | 0.0768 | 86.33/79.74/86.39 | 2.27/2.23/1.54 |
|  | Sheep liver | Y=2872.981x+558.367 | 0.9978 | 0.0503 | 0.0810 | 84.87/87.90/81.29 | 1.33/1.02/0.45 |
| FLE | Cattle muscle | Y=3906x-256.886 | 0.9968 | 0.1288 | 0.2074 | 88.57/94.53/98.11 | 5.16/1.25/1.28 |
|  | Sheep muscle | Y=3969.09x+2610.29 | 0.9978 | 0.1199 | 0.1930 | 87.75/83.36/87.75 | 5.81/1.72/1.11 |
|  | Sheep kidney | Y=3465.23x-527.88 | 0.9956 | 0.1290 | 0.2077 | 91.00/87.50/80.50 | 7.25/2.98/1.29 |
|  | Sheep liver | Y=3717.11x+244.5 | 0.9962 | 0.1323 | 0.2130 | 94.56/91.06/87.56 | 8.69/3.21/2.27 |
| OFL | Cattle muscle | Y=5226.22x-65.9662 | 0.9961 | 0.1423 | 0.3173 | 85.11/85.16/88.32 | 10.67/2.24/0.64 |
|  | Sheep muscle | Y=4865.79x-61.42 | 0.9965 | 0.1456 | 0.3247 | 87.98/84.73/81.47 | 5.68/2.07/0.75 |
|  | Sheep kidney | Y=4685.58x+591.42 | 0.9969 | 0.1566 | 0.3492 | 84.63/87.58/79.53 | 2.07/1.70/1.43 |
|  | Sheep liver | Y=4505.36x+56.867 | 0.9979 | 0.1643 | 0.3664 | 80.18/76.23/79.26 | 9.81/1.33/1.10 |
| SAR | Cattle muscle | Y=2971.73x-197.174 | 0.9993 | 0.0978 | 0.2181 | 84.27/80.43/82.39 | 1.72/2.88/1.34 |
|  | Sheep muscle | Y=2852.86x+1892.87 | 0.9977 | 0.0978 | 0.2181 | 90.27/86.77/88.70 | 3.98/2.10/2.09 |
|  | Sheep kidney | Y=2615.12x+1735 | 0.9956 | 0.1129 | 0.2518 | 90.33/89.74/86.39 | 3.21/1.32/0.57 |
|  | Sheep liver | Y=2377.38x+157.739 | 0.9987 | 0.1231 | 0.2745 | 84.87/80.90/79.29 | 2.77/1.54/1.27 |
| DIF | Cattle muscle | Y=4137.55x+164.282 | 0.9955 | 0.1176 | 0.1682 | 102.52/82.83/100.31 | 6.71/0.84/1.11 |
|  | Sheep muscle | Y=3861.71x+153.33 | 0.9979 | 0.1099 | 0.1572 | 97.71/86.85/93.37 | 6.51/3.09/2.33 |
|  | Sheep kidney | Y=3677.82x+146.028 | 0.9981 | 0.1087 | 0.1554 | 93.74/80.35/81.69 | 7.59/4.42/1.25 |
|  | Sheep liver | Y=3493.93x+138.73 | 0.9973 | 0.1188 | 0.1699 | 93.71/81.09/82.90 | 8.66/2.99/2.18 |
| TC | Cattle muscle | Y=4325.61x+1034.32 | 0.9903 | 0.3184 | 0.4553 | 83.04/82.77/75.64 | 4.37/1.04/0.46 |
|  | Sheep muscle | Y=4543.9x+2165.13 | 0.9933 | 0.3216 | 0.4599 | 87.78/85.41/87.79 | 3.66/2.22/2.11 |
|  | Sheep kidney | Y=3932.37x+940.291 | 0.9963 | 0.3612 | 0.5165 | 84.04/83.77/83.51 | 2.05/2.09/1.56 |
|  | Sheep liver | Y=3539.14x+846.262 | 0.9993 | 0.3889 | 0.5561 | 87.79/85.42/83.80 | 8.07/5.32/3.22 |
| DXC | Cattle muscle | Y=3136.98x+494.13 | 0.9955 | 0.1050 | 0.2625 | 82.63/77.64/83.93 | 2.83/0.22/0.91 |
|  | Sheep muscle | Y=2851.80x+59.06 | 0.9946 | 0.1103 | 0.2758 | 87.80/85.43/82.81 | 6.73/2.82/1.79 |
|  | Sheep kidney | Y=2709.21x+426.75 | 0.9965 | 0.1991 | 0.4978 | 93.63/78.64/80.23 | 5.67/5.64/4.33 |
|  | Sheep liver | Y=2851.80x+449.209 | 0.9943 | 0.1875 | 0.4688 | 87.81/85.44/79.27 | 4.61/3.46/3.41 |
| OTC | Cattle muscle | Y=3480.96x+423.58 | 0.9925 | 0.0186 | 0.0374 | 82.63/78.4/82.17 | 3.55/1.28/1.13 |
|  | Sheep muscle | Y=3352.04x+407.892 | 0.9957 | 0.0178 | 0.0358 | 92.39/90.72/89.32 | 2.49/2.21/1.23 |
|  | Sheep kidney | Y=3223.11x+392.2 | 0.9938 | 0.0202 | 0.0406 | 102.15/93.38/93.03 | 6.42/3.14/1.33 |
|  | Sheep liver | Y=2965.26x+360.827 | 0.9946 | 0.0221 | 0.0444 | 91.91/88.33/87.29 | 3.33/2.07/1.43 |
| CLC | Cattle muscle | Y=2365.35x+719.15 | 0.9926 | 0.1033 | 0.2076 | 81.73/85.54/84.96 | 6.24/2.07/0.38 |
|  | Sheep muscle | Y=2274.38x+691.49 | 0.9936 | 0.1123 | 0.2257 | 83.88/80.57/78.95 | 5.87/0.61/1.79 |
|  | Sheep kidney | Y=2183.40x+663.831 | 0.9954 | 0.1270 | 0.2553 | 76.03/79.84/77.38 | 5.67/1.20/0.92 |
|  | Sheep liver | Y=1819.50x+553.19 | 0.9967 | 0.1307 | 0.2627 | 88.18/87.77/87.63 | 5.48/1.80/1.04 |
| ERY | Cattle muscle | Y=7927.4x+167.67 | 0.9921 | 0.0746 | 0.1940 | 79.32/86.43/78.95 | 6.13/2.40/0.59 |
|  | Sheep muscle | Y=8493.64x+179.646 | 0.9954 | 0.0819 | 0.2129 | 83.672/83.68/82.26 | 3.27/3.00/2.72 |
|  | Sheep kidney | Y=5662.43x+119.764 | 0.9934 | 0.0987 | 0.2566 | 83.62/81.43/87.33 | 5.93/3.60/1.26 |
|  | Sheep liver | Y=5379.3x+113.776 | 0.9943 | 0.1010 | 0.2626 | 83.568/82.79/86.59 | 4.58/4.19/3.99 |
| CLA | Cattle muscle | Y=16851x+777.90 | 0.9992 | 0.2466 | 0.6412 | 110.3/116.24/117.6 | 1.60/2.36/0.37 |
|  | Sheep muscle | Y=15554.77x+698.062 | 0.9993 | 0.2668 | 0.6937 | 92.37/99.88/87.37 | 1.89/1.28/0.67 |
|  | Sheep kidney | Y=14906.65x+688.14 | 0.9990 | 0.3870 | 1.0062 | 94.55/92.93/89.90 | 2.93/1.20/1.53 |
|  | Sheep liver | Y=12962.31x+598.385 | 0.9994 | 0.4110 | 1.0686 | 90.30/85.97/87.40 | 3.97/1.88/1.73 |
| AZI | Cattle muscle | Y=3188.25x-19.70 | 0.9996 | 0.4674 | 1.2152 | 82.88/87.81/78.19 | 6.02/2.61/0.99 |
|  | Sheep muscle | Y=3055.41x-18.88 | 0.9989 | 0.4899 | 1.2737 | 100.18/95.41/90.64 | 8.33/5.02/5.09 |
|  | Sheep kidney | Y=2524.03x+15.596 | 0.9997 | 0.5007 | 1.3018 | 86.15/91.31/86.79 | 5.49/4.48/3.47 |
|  | Sheep liver | Y=2258.34x+13.95 | 0.9992 | 0.5210 | 1.3546 | 80.18/86.23/79.26 | 9.73/3.94/1.85 |
| RTM | Cattle muscle | Y=6241.35x+48.22 | 0.9984 | 0.3869 | 1.0059 | 88.35/85.32/80.12 | 8.02/0.79/2.65 |
|  | Sheep muscle | Y=6085.32x-37.61 | 0.9988 | 0.4236 | 1.1014 | 90.37/88.18/86.00 | 6.31/1.76/1.65 |
|  | Sheep kidney | Y=5773.25x+44.6 | 0.9985 | 0.4765 | 1.2389 | 92.77/90.37/83.02 | 4.59/2.73/0.87 |
|  | Sheep liver | Y=6241.35x+48.22 | 0.9984 | 0.4899 | 1.2737 | 95.55/94.67/96.51 | 2.88/3.71/1.22 |
| TIL | Cattle muscle | Y=1402.02x-57.13 | 0.9976 | 0.5212 | 1.3551 | 90.79/88.17/95.42 | 3.89/4.45/1.71 |
|  | Sheep muscle | Y=1389.98x-66.78 | 0.9978 | 0.4993 | 1.2982 | 88.23/86.70/84.33 | 4.90/5.19/2.20 |
|  | Sheep kidney | Y=1168.35x+476.08 | 0.9981 | 0.5097 | 1.3252 | 93.80/91.76/90.23 | 5.91/5.94/2.69 |
|  | Sheep liver | Y=1215.08x+49.5 | 0.9983 | 0.5199 | 1.3517 | 99.78/87.26/89.42 | 6.92/6.68/3.18 |

Table S3. Mean concentration (μg/kg) of the selected antibiotics (Sulfaquinoxaline, sulfadimethoxine, ofloxacin, sarafloxacin, doxycycline, erythromycin, clarithromycin, azithromycin, roxithromycin and tilmicosin were not detected)

| Antibiotics | | Mean concentration (μg/kg) | | | |
| --- | --- | --- | --- | --- | --- |
|  |  | Cattle muscle | Sheep muscle | Sheep kidney | Sheep liver |
| Sulfonamides | Sulfadiazine | 0.172 | 0.986 | 0.205 | 0.771 |
|  | Sulfamethoxazole | 0.400 | 0.665 | 0.015 | 0.030 |
|  | Sulfamerazine | 0.049 | 0.180 | 0.520 | 1.136 |
|  | Sulfamethazine | 0.072 | 0.064 | 0.329 | 0.550 |
|  | Sulfamonomethoxine | 19.953 | 0.293 | 0.009 | 0.002 |
|  | Trimethoprim^a^ | 1.482 | 0.086 | 0.001 | 0.010 |
| Fluoroquinolones | Norfloxacin | 0.012 | 0.017 | 0.021 | 0.315 |
|  | Enoxacin | 0.126 | 0.013 | 0.479 | 1.481 |
|  | Ciprofloxacin | 0.079 | 0.306 | 0.000 | 0.353 |
|  | Danofloxacin | 0.005 | 0.017 | 0.006 | 0.139 |
|  | Enrofloxacin | 0.014 | 0.053 | 0.029 | 0.053 |
|  | Fleroxacin | 0.100 | 0.000 | 0.000 | 0.021 |
|  | Difloxacin | 0.011 | 0.138 | 0.000 | 0.017 |
| Tetracyclines | Tetracycline | 0.027 | 0.238 | 0.170 | 0.148 |
|  | Oxytetracycline | 0.863 | 0.029 | 0.013 | 0.030 |
|  | Chlortetracycline | 0.491 | 1.350 | 3.406 | 3.828 |

a: Due to being usually used with sulfonamides in practice, trimethoprim was combined with sulfonamides

Table S4. Estimated daily intake values (EDI) and percentage of EDI to acceptable daily intake (ADI) ratio using averages and worst case scenario (wcs) approaches (sulfaquinoxaline, sulfadimethoxine, ofloxacin, sarafloxacin, doxycycline, erythromycin, clarithromycin, azithromycin, roxithromycin and tilmicosin were not detected)

| Antibiotics | EDI (Mean) (ng/kg bw/day) | EDI (wcs) (ng/kg bw/day) | %EDI to ADI Ratio (Mean) | %EDI to ADI Ratio (wcs) |
| --- | --- | --- | --- | --- |
|  |  |  |  |  |
| **Sulfonamides** | 6.595 | 132.656 | 0.02186 | 0.44109 |
| Sulfadiazine | 0.678 | 12.757 | 0.00136 | 0.02551 |
| Sulfamethoxazole | 0.519 | 6.433 | 0.00104 | 0.01287 |
| Sulfamerazine | 0.139 | 2.178 | 0.00028 | 0.00436 |
| Sulfamethazine | 0.065 | 1.011 | 0.00013 | 0.00202 |
| Sulfamonomethoxine | 4.797 | 102.218 | 0.00959 | 0.20444 |
| Trimethoprim^a^ | 0.397 | 8.060 | 0.00946 | 0.19190 |
| **Fluoroquinolones** | 0.445 | 6.507 | 0.00158 | 0.03287 |
| Norfloxacin | 0.015 | 0.131 | - | - |
| Enoxacin | 0.050 | 0.553 | - | - |
| Ciprofloxacin | 0.216 | 2.645 | - | - |
| Danofloxacin | 0.013 | 0.137 | 0.00006 | 0.00069 |
| Enrofloxacin | 0.038 | 0.719 | 0.00061 | 0.01160 |
| Fleroxacin | 0.023 | 0.264 | - | - |
| Difloxacin | 0.091 | 2.059 | 0.00091 | 0.02059 |
| **Tetracyclines** | 1.425 | 25.072 | 0.00475 | 0.08357 |
| Tetracycline | 0.162 | 3.198 | 0.00054 | 0.01066 |
| Oxytetracycline | 0.218 | 3.976 | 0.00073 | 0.01325 |
| Chlortetracycline | 1.045 | 17.899 | 0.00348 | 0.05966 |

-, not calculated (for some ADI values that were unavailable, some values of % EDI to ADI ratio didn’t calculated.); a: Due to being usually used with sulfonamides in practice, trimethoprim was combined with sulfonamides.
